# Douglas Moray Cooper Lamb Argyll Robertson (1837–1909)

**DOI:** 10.1007/s00415-015-7872-7

**Published:** 2015-08-08

**Authors:** Andrzej Grzybowski, Jarosław Sak

**Affiliations:** Department of Ophthalmology, Poznań City Hospital, ul. Szwajcarska 3, 61-285 Poznań, Poland; Chair of Ophthalmology, University of Warmia and Mazury, Olsztyn, Poland; Department of Ethics and Human Philosophy, Medical University of Lublin, Staszica 4/6,102 (Collegium Maximum), 20-059 Lublin, Poland

**Fig. 1:**
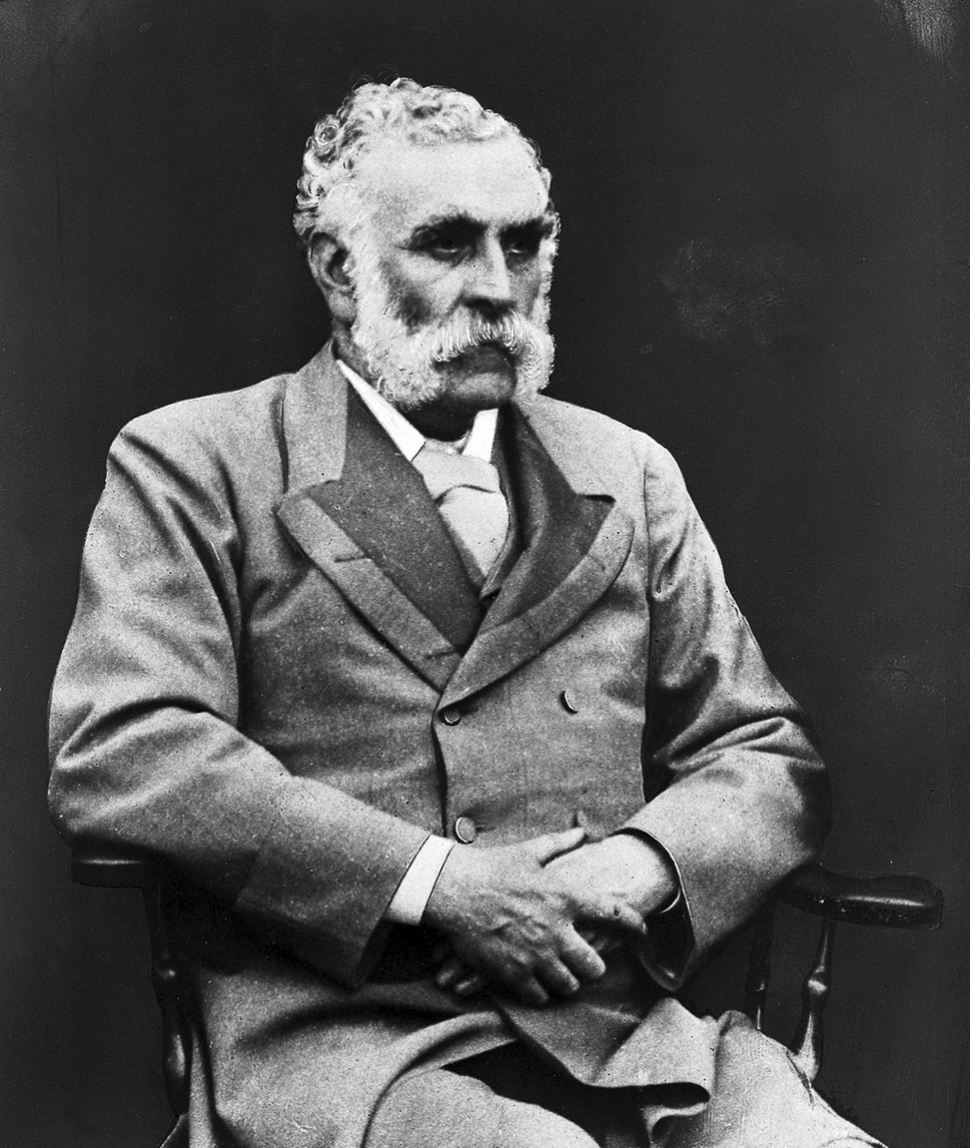
Douglas Argyll Robertson. Photograph. Wellcome Library, London

Douglas Moray Cooper Lamb Argyll Robertson (Fig. 1) was a surgeon–oculist who contributed to the development of neurology by describing Argyll Robertsons pupil [1, 2] and discovering the effects of the Calabar bean (*Physostigma venenosum*) [3].

Robertson was born in Edinburgh, Scotland in 1837. He received his early education in Edinburgh where he began his medical studies. His father and two uncles were Fellows of the Royal College of Surgeons of Edinburgh [4, 5]. He graduated from St. Andrews in 1857 after a 4-year course when he was 20 years old [6]. In the same year, he took the Licenciate of the Royal College of Surgeons of Edinburgh. He studied under von Arlt in Prague and Albrecht von Graefe in Berlin.

After serving as a house surgeon at the Edinburgh Royal Infirmary by 1866 he became full ophthalmic surgeon. In 1886, he was elected President of the Royal College of Surgeons of Edinburgh and possessing a courteous manner and a fluency in both French and German, he was asked to preside over ophthalmic congresses held in Lucerne and Utrecht. He was the first surgeon outside London to be chosen President of the Ophthalmological Society of the United Kingdom in 1893–1895. In 1896, he received degree of LL.D. from the University of Edinburgh. He was honorary surgeon–oculist both to Queen Victoria and King Edward VII.

Argyll Robertson was a very good golfer. He regarded this game as the best recreation [6]. In 1904, for health reasons, he moved to a farm in St. Aubyns on the Isle of Jersey. He caught a cold and died at Gonday near Bombay on 3rd January 1909, while on his third visit to India. His body was cremated on the bank of the river Gondli [4–7, 9].

Argyll Robertson had broad medical interests, and emphasized the role of ophthalmology in a wider medical context. He lectured on the topic of “The therapeutical contributions of ophthalmology to general medicine” [8] at his inauguration as President of the Ophthalmological Society. According to his obituary he “preferred the tongue to the pen as a medium” [7]. He published more than 50 medical papers and communications on neuroophthalmology, anatomy and physiology of eye, treatment of cataract and on the etiology of glaucoma, retinitis pigmentosa, and albuminuretic retinopathy.

In 1863 in the *Edinburgh Medical Journal* [3] Argyll Robertson reported the ocular effects of the Calabar bean (*Physostigma venenosum*) which is the seed of a leguminous plant found in Calabar, in the eastern region of Nigeria. A solution of the seed extract was used by the natives for judicial execution. The active agent of the Calabar bean is physostigmine, a cholinesterase inhibitor. Although it was Sir Robert Christison (1797–1882), one of the Argyll Robertson’s teachers, who reported the first experimental use of the bean, he did not describe any effects on the eye but recommended its use for the humane execution of criminals [5, 6, 9]. Argyll Robertson dropped an extract into his own eye and made the deduction that physostigmine contracts the pupil. He described this experiment:

On the 17th of January, I carefully examined the condition of my eyes, and found that both my sight was normal. (…) I introduced a drop of the weakest extract of the Calabar bean into my left eye (…). At 12:30, or 20 min after the introduction of the extract, a marked alteration in the size of the pupils was observable; the left pupil being only 1 line in diameter, while the right measured fully two lines. (…) These experiments prove that the local application of the Calabar bean to the eye induces—first, a condition of shortsightedness (…). And second, it occasions contraction of the pupil, and sympathetically dilatation of the pupil of the other eye [3].

Argyll Robertson showed the antagonistic property of the Calabar bean to atropine, and this agent became the first effective medication to treat glaucoma.

In 1869, he described symptoms of tertiary syphilis of the nervous system concerning pupils (absence of the light response and brisk accommodation reaction) in two articles [1, 2]. In February 1869, Robertson described a case report of a 59-year-old patient affected with central nervous system syphilis, admitted on account of an unsteady gait due to spinal disease [1]. In the second article, in December 1869, he published four more similar cases [2]. In the first paper, he wrote: “I could not observe any contraction of either pupil under the influence of light, but, on accommodating the eyes for a near object, both pupils contracted.” [1] Although the absence of pupillary light response in patients with spinal disease had been reported previously, Argyll Robertson was the first to realize that the pupils still reacted to near stimuli [9]. This relationships between unreactivity to light and preserved accommodation reaction had been reported earlier by a few physicians. In 1793, the Italian psychiatrist Vincenzo Chiarugi (1759–1820), in 1840 Moritz Heinrich Romberg (1795–1873), and Albrecht von Graefe (1828–1870) in 1856 mentioned this combination of signs [5, 9, 10]. They noted that in certain cases of spinal disease the pupils were small with absent reaction to light but did not analyze this relationship. Robertson was the first who tried to explain the etiology of this syndrome. He suspected that the responsible lesion could be found in the sympathetic system of cervical spinal cord. He indicated that destruction of the sympathetic fibers would suffice to explain both the absent light reflex and miosis [5]. Although for many years Argyll Robertson pupil was a sign of tertiary syphilis, it is known today that it might be seen in other disorders, including diabetic neuropathy, Parinaud syndrome, brain injury, thiamine deficiency, and mitral regurgitation.
